# Protocol of a Randomized Trial Investigating the Value of a Reminder App to Increase Physical Activity During Radiotherapy or Radio-Chemotherapy for Locally Advanced Lung Cancer

**DOI:** 10.3390/jcm15145678

**Published:** 2026-07-20

**Authors:** Dirk Rades, Maria Karolin Streubel, Liesa Dziggel, Cansu Delikanli, Sabine Bohnet, Stefan Janssen, Darejan Lomidze, Jon Cacicedo, Jasna But-Hadzic, Hanne Falk Grauslund, Charlotte Kristiansen, Christian Felix Schulz

**Affiliations:** 1 Department of Radiation Oncology, University of Luebeck, 23562 Luebeck, Germany; stefan.janssen@uksh.de; 2 Department of Radiation Oncology, University Medical Center Schleswig-Holstein, Luebeck Campus, 23538 Luebeck, Germany; mariakarolin.streubel@uksh.de (M.K.S.); liesa.dziggel@uksh.de (L.D.); cansu.delikanli@uksh.de (C.D.); 3 Department of Pulmonology, University of Luebeck, 23562 Luebeck, Germany; sabine.bohnet@uksh.de; 4 Department of Pulmonology, University Medical Center Schleswig-Holstein, Luebeck Campus, 23538 Luebeck, Germany; 5 MVZ RON Niedersachsen Strahlentherapie GmbH, 30161 Hannover, Germany; 6 Radiation Oncology Department, Tbilisi State Medical University, 0177 Tbilisi, Georgia; dlomidze@hotmail.com; 7 Radiation Oncology Department, Ingorokva High Medical Technology University Clinic, 0177 Tbilisi, Georgia; 8 Department of Radiation Oncology, Cruces University Hospital, 48903 Barakaldo, Spain; jon.cacicedofernandezbobadilla@osakidetza.eus; 9 Department of Radiation Oncology, Biobizkaia Health Research Institute, 48903 Barakaldo, Spain; 10 Department of Radiotherapy, Institute of Oncology Ljubljana, 1000 Ljubljana, Slovenia; jbut@onko-i.si; 11 Department of Oncology, Faculty of Medicine, University of Ljubljana, 1000 Ljubljana, Slovenia; 12 Department of Oncology, Vejle Hospital, University Hospital of Southern Denmark, 7100 Vejle, Denmark; hanne.falk.grauslund@rsyd.dk (H.F.G.); charlotte.kristiansen@rsyd.dk (C.K.); 13 Department of Radiation Oncology, Christian-Albrechts University Kiel, 24105 Kiel, Germany; christian.schulz@uksh.de; 14 Department of Radiation Oncology, University Medical Center Schleswig-Holstein, Kiel Campus, 24105 Kiel, Germany

**Keywords:** lung cancer, radiotherapy, systemic treatment, physical activity, step count, reminder app

## Abstract

**Background**: Radiation therapy with or without systemic treatment for lung cancer can cause adverse reactions, e.g., fatigue. Affected patients may experience a decrease in physical activity and, as a consequence, be unable to receive the complete assigned treatment. Physical exercise may be helpful. A benefit of exercise regarding completion of systemic treatment and quality of life was previously suggested. Irradiated patients may benefit as well. A smartphone-based app reminding patients to make a minimum number of steps might positively influence physical activity. The prototype of an app called “Step Reminder” was recently finalized. The randomized APPAREL trial (NCT07627022) evaluates whether this app can improve physical activity during irradiation for lung cancer. **Methods**: The primary endpoint of this phase 2 trial focuses on the within-patient change regarding the mean number of daily steps (week 5 minus week 1). Secondary objectives include satisfaction with the application and its impact on the perception of digital health technology. Patients are randomized 1:1 to be treated with a standard approach (conventionally fractionated irradiation with or without systemic treatment) supported by the “Step Reminder” app (Arm A) or standard treatment without the app (Arm B). The app provides reminders several times per day to walk a pre-defined number of steps. To make sure that the required 28 patients are included in the primary analysis population (full analysis set), 32 patients have to be randomized. **Results**: At this stage, results are not available. **Conclusions**: It is expected that the app will have a positive effect on the patients’ physical activity (number of steps).

## 1. Introduction

Conventionally fractionated radiation therapy is a frequently applied treatment for non-small-cell (NSCLC) or small-cell (SCLC) lung cancer [1,2]. This type of treatment might cause side effects within the radiation field or general symptoms such as fatigue, resulting in a decreased level of physical activity or function [3,4,5,6,7,8]. In case of more severe treatment-related side effects, the patients may be unable to receive the complete treatment they have been assigned to. Maintaining or improving the level of physical activity by any kind of physical exercise may be of benefit.

Such an effect was shown for cancer patients receiving neoadjuvant chemotherapy, of whom 16.8% (31 patients) had lung cancer [9]. Patients undergoing an exercise program could receive chemotherapy more frequently. In other studies, physical activity resulted in improvement of quality of life in patients with lung cancer [10,11,12,13,14,15,16,17,18]. In a retrospective study of 50 patients receiving chemotherapy for advanced or recurrent lung cancer, a lower level of activity was negatively associated with survival [19]. According to these studies, physical activity appears important for patients treated for lung cancer. Exercise programs can be challenging for the patients, particularly if their well-being is affected by fatigue or other adverse reactions [20,21].

It would be interesting to know whether, in addition to or as an alternative to in-person group training programs, a mobile application that reminds patients to walk a certain number of steps will have a positive impact on physical activity. The prototype of a corresponding app called “Step Reminder” was recently finalized. This randomized phase 2 trial (APPAREL) is going to evaluate whether the “Step Reminder” app can help improve physical activity during a series of radiation therapy with or without systemic treatment administered for locally advanced lung cancer. Improvement is defined as a reduction in the difference in the average number of daily steps (week 5 minus week 1) during the period of radiation treatment.

## 2. Materials and Design

### 2.1. Objectives

The primary objective is the exploration of whether the “Step Reminder” app will lead to alteration of the physical activity during a course of irradiation. The primary endpoint is the within-patient alteration of physical activity, given by the weekly average number of steps per wear-hour of the smartphone, between week 1 and week 5. Study groups A and B are compared to identify potential differences due to the support of the “Step Reminder” app. This endpoint represents a normalized activity metric and is commonly applied in digital health studies to account for heterogeneity in device usage and to reduce bias associated with unequal observation periods across patients [22]. It is a behavioral digital activity endpoint selected because the intervention directly aims to maintain or increase physical activity during radiotherapy. It is not considered a validated surrogate endpoint for treatment completion, toxicity reduction, quality of life, or survival. The potential clinical relevance is supported indirectly by prior studies suggesting associations between physical activity/exercise and quality of life, treatment tolerance, hospitalization risk, and prognosis in lung cancer and other oncology populations; however, causal conclusions on these outcomes are beyond the scope of the present protocol. Secondary objectives include patient satisfaction with the “Step Reminder” app and the potential influence of using this app on the patient’s attitude towards digital health technology (assessed at the end of week 5). Furthermore, exploratory analyses investigate whether the observed effects are different between baseline characteristic-related subgroups. Due to the limited sample size, these analyses can only help to generate hypotheses and inform the design of future studies.

### 2.2. Design and Duration

This is a multicenter prospective randomized study (phase 2) in patients irradiated for lung cancer. Patients are randomized 1:1 and receive standard irradiation and the “Step Reminder” app (Arm A) or standard irradiation without the “Step Reminder” app (Arm B). “Step Reminder” is a smartphone-based application that provides three or four reminders per day (patient’s selection) to motivate the patients to walk a pre-defined number of steps each day. Randomization will be stratified by concurrent systemic therapy [23].

Physical activity is going to be assessed at previously defined time points during the period of radiation therapy, i.e., during week 1 and week 5, using step count data from an integrated step counter of the smartphone or a stand-alone device. The recruitment of the required 32 patients will be finalized within 6 months. Each patient will stay in the study for the radiotherapy course, which takes 5–7 weeks. Thus, overall study time is expected to be 8 months.

The criteria required to qualify for inclusion in the APPAREL trial include lung cancer (confirmed by pathology), treatment with at least 50 Gy of conventionally fractionated radiation therapy, and availability of a smartphone with an already integrated step counter or a smartphone supplemented by a corresponding health app. Patients must be 18 years or older and provide written informed consent. Patients unable to consent or giving the impression of insufficient compliance may not be included.

Patients can terminate their participation in the trial at any time without negative consequences. Participation may be stopped prematurely by the principal investigator if reasonably justified. In this situation, the affected patient and the ethics committee will be informed promptly.

### 2.3. Registration and Randomization

The patient code consists of the number of the contributing center (one digit), followed by a letter for the treatment approach (R = radiotherapy alone, S = radiotherapy plus systemic treatment) and a consecutive number consisting of 3 digits. Example: 1R-001.

Patients will be randomized 1:1 to receive either standard treatment for lung cancer supported by the “Step Reminder” app (Arm A) or standard treatment for lung cancer without the app (Arm B) using a block randomization scheme. Randomization is stratified according to presence or absence of concurrent systemic therapy (yes vs. no) [23]. The randomization list is going to be provided by an external professional statistician. The information regarding randomization (Arm A or Arm B) will be obtained from the department of the principal investigator. Study personnel will provide standardized instructions regarding smartphone handling to all participants to ensure consistent data collection conditions across study sites.

### 2.4. Procedures

Data assessed prior to radiation therapy include medical history, medication, demographics, body mass index, Karnofsky performance score, National Comprehensive Cancer Network (NCCN) distress thermometer (including physical, emotional, social, practical, and spiritual or religious concerns [24,25]), pain score (patient self-assessment, points from 0 to 10), fatigue (patient self-assessment, points from 0 to 10), tumor histology, tumor stage, systemic treatment, parameters of the planned radiation therapy, and the number of daily reminders (three or four) the patient has chosen. Assessments performed at the time of screening, at baseline, and during radiation therapy are given in Table 1.

#### 2.4.1. Radiation Therapy

Patients receive a standard treatment for lung cancer according to the current German guideline [2]. For patients with inoperable locally advanced NSCLC, the treatment often includes fractionated radiation therapy with total doses of 66–70 Gy (dose per fraction = 2.0 Gy) conventionally. It may be combined with concurrent chemotherapy. Depending on the histologic subtype, cisplatin is combined with vinorelbine (squamous cell carcinoma), pemetrexed (adeno-carcinoma), or paclitaxel (both subtypes), followed by consolidation therapy with durvalumab [2]. Some patients with SCLC may be irradiated with daily fractions of 2.0 Gy up to 60–66 Gy. For these patients, radiation treatment is generally combined with four to six courses of chemotherapy, of which two courses are given concurrently. Standard chemotherapy for SCLC includes cisplatin and etoposide [2]. For patients not experiencing progression during radio-chemotherapy, consolidation systemic treatment with durvalumab may be offered. Irradiation may be associated with acute or subacute side effects, which may cause an interruption of the treatment. If the next radiation fraction is delayed for more than 7 days, the principal investigator has to be notified.

#### 2.4.2. Number of Daily Steps

Physical activity will be documented under pragmatic real-world conditions using daily step counts recorded by an already integrated step counter of a patient-owned smartphone or a corresponding health app downloaded prior to the start of radiation therapy. Data are received from the patient’s smartphone for weeks 1 and 5. This pragmatic approach may introduce variability between devices and applications; however, the primary endpoint is based on within-patient change, which partially mitigates between-device differences. Data are extracted by trained members of the trial group to make sure the recorded information is complete and consistent.

To ensure standardized carrying behavior, patients will be instructed to carry their smartphone continuously during waking hours whenever feasible, irrespective of whether they are physically active or not. For each valid day, steps per wear-hour are obtained by dividing the total number of daily steps by the wear-time per day (hours). For a valid day, the wear-time must be 4 h or longer. Daily normalized step counts will be aggregated into weekly averages based on all valid days in the respective week. To be included in the analyses, the corresponding week (week 1 or 5) must include at least four valid days. The wear-time will be obtained from the patient’s smartphone.

#### 2.4.3. “Step Reminder” App

The “Step Reminder” app has been made and will be maintained by Nextlabel Offene Handelsgesellschaft (OHG), Luebeck, Germany. It reminds the patients three or four times per day (patient’s choice) to walk a certain number of steps during the radiotherapy course. In addition, the “Step Reminder” app will record the number of reminders sent per day, the timestamp of each reminder, and any potential user interaction. These parameters will be used as exploratory measures of adherence to the intervention. Nextlabel OHG will also provide the required documentation for the “Step Reminder” app. All rights of the “Step Reminder” app remain with the developing company. For individual activation of the app, the company requires the patient’s e-mail address. To ensure its protection, the sponsor of the APPAREL trial, the University Medical Center Schleswig-Holstein, and the developer of the “Step Reminder” (Nextlabel OHG) app entered into a contract including an approved data protection concept. The sponsor and Nextlabel OHG have neither a role in designing the APPAREL trial nor in handling the data. Nextlabel OHG is involved in the Interreg project Health-Advancing Technology for the Elderly as a project partner, but its role is limited to the production of mobile applications.

#### 2.4.4. Patient Satisfaction with the “Step Reminder” App

Prior to the start of radiotherapy, patients are asked how much they are used to handling a smartphone and whether they require assistance regarding the installation of a health app or the “Step Reminder” app. At the end of week 5, patients randomized to Arm A complete a questionnaire focusing on satisfaction with the app and a potential change of “digital behavior”, for example, with respect to using the internet or other apps to enquire about different aspects of their disease [26,27]. If less than 80% of the patients are satisfied with the “Step Reminder” app, it should be further optimized. If less than 60% of the patients are satisfied, the app is not suitable for the purpose assessed in the APPAREL trial and should be further optimized. The 80% and 60% thresholds are predefined feasibility and acceptability criteria for app optimization. They represent pragmatic operational definitions rather than evidence-based cut-off values and are not statistically derived cut-offs. A satisfaction rate below 80% indicates room for further optimization before broader use, whereas a satisfaction rate below 60% indicates insufficient acceptability for the intended purpose.

#### 2.4.5. Adverse Events

Adverse reactions are rated according to the Common Terminology Criteria for Adverse Events classification 5.0 [28]. Serious and unexpected events have to be indicated within 24 h after their detection to the principal investigator.

### 2.5. Statistics

#### 2.5.1. Sample Size

This prospective randomized study is designed to analyze the effect of the “Step Reminder” app on physical activity during irradiation in lung cancer patients. The primary endpoint is the within-patient change in weekly average steps per wear-hour between the fifth and the first week. The primary analysis includes all randomized patients with evaluable data regarding the primary endpoint. The comparison (“Step Reminder” app group vs. control group) is made with a two-sided Wilcoxon–Mann–Whitney U test.

Calculation of the required sample size considers a clinically relevant difference corresponding to a relative difference in the within-patient change in steps per wear-hour of about 30% between treatment arms. Given the non-parametric nature of the primary analysis, this assumption is interpreted as a moderate shift in the distribution of the endpoint, which represents a probability of superiority of about 0.65. The assumed 30% relative difference represents a pragmatic planning assumption for this exploratory randomized phase 2 trial and should not be interpreted as an established minimal clinically important efficacy difference for this endpoint. Assuming a two-sided significance level of α = 0.05 and a statistical power of 80%, 28 patients are needed to detect such an effect using the asymptotic approximation of the Wilcoxon–Mann–Whitney U test. It is assumed that approximately 10% of the randomized patients cannot be evaluated with respect to the primary endpoint. Therefore, 32 patients need to be randomized. The calculation aligned with the analysis population, and the endpoint definition is considered appropriate for this exploratory study. The multicenter design primarily supports recruitment feasibility and broader applicability of the exploratory findings; it is not intended to imply a confirmatory multicenter efficacy trial. Results are interpreted descriptively and will contribute to the design of additional larger studies.

#### 2.5.2. Statistical Aspects

The full analysis set includes all randomized patients who provide evaluable data for the primary endpoint. Evaluable data are defined as at least one valid week of step count data, i.e., a week with at least four valid days (valid day = at least 4 h of smartphone wear-time). Patients without evaluable data in either week 1 or week 5 will be excluded from the primary endpoint analysis. This definition ensures a reliable estimation of weekly average steps per wear-hour and is considered appropriate considering the nature of the digital endpoint and the exploratory design of the APPAREL trial. The full analysis set is not intended to represent a strict intention-to-treat population; it includes randomized patients with evaluable primary endpoint data. The number of randomized patients excluded from the full analysis set and the reasons for exclusion will be reported by treatment arm. A Consolidated Standards of Reporting Trials (CONSORT) flow diagram will be provided explicitly reporting the number of randomized patients excluded from the primary analysis, along with detailed reasons for exclusion in each treatment arm.

The per-protocol set is defined as a subset of the full analysis set, including patients without major protocol deviations, with adequate adherence to study procedures, and with sufficient and consistent data quality. In addition to meeting these criteria, patients of the per-protocol set must demonstrate adequate compliance with smartphone use and adherence to the assigned treatment. For Arm A, this means documented use of the “Step Reminder” app during the majority (≥80%) of the first five weeks of the radiation therapy period. For Arm B, this means no use of any comparable reminder app or structured activity intervention.

Statistical analysis is based on observed cases only. Patients must meet the predefined criteria for valid data in week 1 and week 5 to be included in the primary analysis. Missing data, if any, may be expected to be not missing completely at random, particularly in relation to insufficient smartphone wear time. Therefore, the extent and patterns of missing data will be described descriptively, and reasons for non-evaluable data will be summarized. Because of the small sample size and the exploratory nature of the APPAREL trial, no formal imputation and no best-case, worst-case, or model-based missing-data sensitivity analyses will be performed. The predefined approach for missing primary endpoint data is restricted to complete-case analysis, and the results are interpreted in light of the observed missing data structure. The per-protocol set is analyzed using the same statistical methodology as for the primary analysis. Results from the per-protocol set are considered supportive and will be used to assess the consistency and robustness of the findings in the full analysis set. Statistical tests are performed as two-sided tests with a nominal significance level of α = 0.05. Given the exploratory nature of this randomized trial, no adjustment for multiplicity is applied, and *p*-values beyond the primary endpoint analysis are interpreted descriptively. All secondary, subgroup, adjusted, and sensitivity analyses will be interpreted descriptively. Nominal *p*-values from these analyses will not be interpreted as confirmatory.

All available data will be used irrespective of adherence to the assigned treatment, including non-use or discontinuation of the reminder app, provided that sufficient data are available for endpoint calculation. Exploratory adjusted analyses may be performed to account for potential baseline imbalances, including baseline physical activity and the stratification factor (concurrent systemic treatment: yes vs. no). These analyses can be interpreted as supportive. Fatigue, pain, distress, and performance status will be summarized descriptively and may be explored in supportive analyses to contextualize changes in physical activity. They are not included as mandatory covariates in the primary model due to the small sample size and the risk of overfitting.

#### 2.5.3. Statistical Aspects Related to the Primary Endpoint

Primary analysis is performed in the full analysis set and compares the distribution of within-patient changes between the treatment groups (“Step Reminder” app vs. control). No imputation of missing data is performed. The analysis is based on observed cases only. Extent and pattern of missing data are described descriptively. The primary estimand is directed at the difference between treatment groups in the average within-patient change in physical activity between the fifth and the first week. Intercurrent events, including incomplete smartphone use, technical failures, or treatment interruptions, are addressed using a treatment policy strategy, where available, valid data are included without imputation. Descriptive statistics of the primary endpoint will be provided by treatment group, including the number of subjects with evaluable data, mean (plus standard deviation), and median (plus quartiles, minimum, and maximum). Individual patient trajectories are visualized using spaghetti plots. The endpoint represents a normalized activity metric and is intended to account for heterogeneity in device usage and to reduce bias associated with unequal observation periods. Wear-time has been shown to influence estimates derived from consumer-grade wearable data, supporting the need to account for device usage time in digital physical activity analyses.

The primary comparison between treatment groups is performed with the two-sided Wilcoxon–Mann–Whitney U test. The magnitude of the treatment effect is estimated using the Hodges–Lehmann estimator together with a two-sided 95% confidence interval. Given the limited sample size, the results are interpreted descriptively only. A paired *t*-test may be reported as an additional analysis if the distribution of within-patient differences is approximately normal.

Within each treatment group, the change between week 1 and week 5 is analyzed using the Wilcoxon signed-rank test. A paired *t*-test may be provided as a supportive analysis if appropriate. If diagnostic checks indicate substantial skewness, a supportive analysis on the log scale is performed, and results are expressed as ratios of geometric means with corresponding 95% confidence intervals, without changing the primacy of the analysis on the original scale.

As a further exploratory supplementary analysis, a linear mixed-effects model is fitted, including fixed effects for time (week 1, week 5), treatment (“Step Reminder” app vs. control), and their interaction, as well as a random intercept for the patient. The treatment-by-time interaction represents the difference between treatment groups in the change from week 1 to week 5. Results from this model are reported as point estimates, two-sided 95% confidence intervals, and descriptive *p*-values. They have to be interpreted cautiously due to the limited sample size. If appropriate, a log-transformed analysis is provided as a sensitivity analysis.

Exploratory subgroup analyses may be conducted to assess whether the treatment effect differs across subgroups, namely age category, performance status, histology, concurrent systemic treatment, and type of smartphone (iOS vs. Android). Given the small overall sample size and the resulting low numbers within subgroups, these analyses can only be regarded as exploratory and hypothesis-generating.

#### 2.5.4. Statistical Aspect Related to Secondary Endpoints

Secondary endpoints are analyzed in an exploratory and descriptive manner, and results are interpreted cautiously in light of the limited sample size. Satisfaction with the “Step Reminder” app is evaluated with a structured questionnaire at the end of week 5 (Arm A only). Individual questionnaire items are summarized using appropriate descriptive statistics. Categorical responses (e.g., Likert scale) are presented as absolute and relative frequencies, while continuous or score-based measures are summarized using mean (standard deviation) and median (quartiles, range). An overall dissatisfaction rate may be calculated based on predefined criteria and presented together with corresponding 95% confidence intervals. The analysis aims to provide a descriptive assessment of usability and acceptance of the app. The analysis of the impact of the “Step Reminder” app on use and perception of digital health technologies is based on questionnaire data collected at week 5 as well. The results are summarized descriptively, and composite scores may be calculated where appropriate. Satisfaction with the application and impact on the use of digital health technology are descriptive feasibility and acceptability outcomes. No formal inferential hypotheses are planned for these outcomes.

In addition, exploratory analyses may assess whether effects vary in subgroups related to baseline characteristics such as age, performance status, NCCN distress thermometer (including physical, emotional, social, practical, and spiritual or religious concerns [24,25]), pain score, fatigue, prior experience with smartphones, or the number of daily reminders the patient has selected. Given the limited number of subjects per subgroup, the results must be interpreted with caution.

### 2.6. Data Management and Dissemination Plans

The APPAREL trial does not require additional medical procedures that may be considered potentially burdensome for the patient. The APPAREL trial is performed following the Declaration of Helsinki and the Data Protection Act. Data related to this trial are handled with the highest confidentiality. Precautionary measures are undertaken to prevent the data from being communicated to unauthorized individuals. Patients can be identified solely by their personal ID code. The patients who are randomized to Arm A and, thus, assigned to use the “Step Reminder” app must give consent that their e-mail address will be forwarded to Nextlabel OHG, from where they will receive a link to download the app. For appropriate data protection, Nextlabel OHG and the sponsor of the APPAREL trial have already signed a contract including a concept of data protection. The trial protocol, the patient information, and the informed consent form were submitted to and approved by the responsible ethics committee. Prior to being included in the APPAREL trial, each patient is sufficiently informed about the aims, nature, and procedures of the trial and the right to stop participation at any time without stating the reason. The patients are informed that the collected data are documented in a CRF and forwarded for statistical analyses.

The principal investigator, the involved external statistician, and other members of the trial team write a report. Key documents of the APPAREL trial are stored at the sponsor’s institution for a minimum of 10 years after submission of this report. An article reporting the findings of the APPAREL trial is submitted to a peer-reviewed journal. The draft of the corresponding manuscript is reviewed by the external statistician.

## 3. Discussion

Radiation therapy or radio-chemotherapy for locally advanced lung cancer can lead to significant fatigue and a subsequent decrease in physical activity [20,21]. This process may have a remarkable negative effect on the patient’s quality of life and treatment outcomes [9,10,11,12,13,14,15,16,17,18,19]. These impairments may be alleviated by means of regular physical exercise before and during the phase of treatment [8,9,10]. However, when significantly stressed by anticancer treatment and its toxicity, both motivation and ability to regularly perform training sessions and workouts may be limited. Promising approaches to improve this situation include participation in group exercise or yoga programs and physiotherapy. However, depending on the patient’s place of residence and potential waiting lists, these options may not always be available when needed. One can imagine that an app installed on a patient’s smartphone, reminding the patient daily to perform some physical exercise or to walk a minimum number of steps, could also be a valuable support. Such an app called “Step Reminder” is already available but has not yet been tested in patients. In the field of radiation therapy, apps are becoming increasingly popular to support patients [29,30,31,32]. The clinical value of the “Step Reminder” app is evaluated in patients irradiated for lung cancer in the current APPAREL trial. Ideally, this randomized trial will show that the “Step Reminder” app has a positive effect on the patient’s average step count during the period of treatment. However, when interpreting the results of the APPAREL trial after its completion, the corresponding limitations have to be considered. Major limitations include the exploratory nature of the trial and the comparably small sample size of 32 patients. Due to the limited sample size, analyses aiming to determine whether effects are different between subgroups related to baseline characteristics, number of daily reminders chosen by the patients, pain, fatigue, and items addressed in the NCCN distress thermometer [27,28] can only contribute to the generation of hypotheses and to designing future prospective trials. Another important limitation is the fact that suitability for the APPAREL trial includes smartphone ownership and sufficient digital literacy. Therefore, the study may not represent many elderly or frailer patients irradiated for lung cancer. Further limitations include the open-label study design, device and application heterogeneity, potential missing-not-at-random endpoint data, attrition bias due to evaluability criteria, assessment of activity only during weeks 1 and 5, and the possibility of contamination in the control arm through independent use of activity apps or wearable devices. Blinding was considered not feasible in this pragmatic phase II setting, because the intervention consists of active use of a reminder app. The primary endpoint is based on automatically recorded step counts rather than investigator-rated activity, which reduces but does not eliminate behavioral bias. The control arm receives standard care without an app; potential performance effects and increased attention in the intervention arm will be considered when interpreting the results. Moreover, since participants in the intervention arm receive repeated reminders throughout treatment, behavioral changes may partly reflect increased attention (Hawthorne effect) rather than the intrinsic efficacy of the reminders themselves. Unfortunately, it is not possible to eliminate the possibility of this effect in the APPAREL trial.

One has to be aware that other important prognostic variables, such as age, performance status, baseline physical activity, histology, disease burden, and type of smartphone, are not incorporated into the primary analysis. Concurrent systemic treatment was selected as the only randomization stratification factor, since it is expected to have a strong impact on fatigue, toxicity, and physical activity, and since additional stratification factors appear impractical and potentially unstable with only 32 randomized patients. Other baseline characteristics, including age, performance status, histology, distress, pain, and fatigue, will be summarized descriptively and evaluated in exploratory analyses only. One has to be aware that the effect size of 30% has been chosen after discussion with experienced radiation oncologists, medical oncologists, and pulmonologists, but is not supported by clinical evidence. No validated minimal clinically important difference currently exists for normalized step-count endpoints in patients receiving thoracic radiotherapy.

The question may arise why the step counts are limited to weeks 1 and 5. These weeks were selected pragmatically to compare early-treatment activity with activity near the end of conventionally fractionated radiotherapy, while minimizing the burden for the patients and staff members. Due to this approach, intermediate fluctuations will not be captured, which limits the interpretation of temporal trajectories. Satisfaction and digital health adoption outcomes are secondary descriptive endpoints only; they are intended to assess usability, acceptability, and feasibility, not to test formal inferential hypotheses.

Thus, when the results of the APPAREL trial become available, they need to be interpreted with caution due to the above-mentioned limitations and should be mainly considered hypothesis-generating. Moreover, one needs to be aware that in case of positive results, the APPAREL trial can only show that the “Step Reminder” app will contribute only to maintaining or increasing the average weekly step count. Currently, there is no sufficient evidence that an improvement in step counts will translate into better survival or treatment tolerance. The only study suggesting a corresponding survival benefit was a small retrospective study of 50 patients [19]. An association between completing an exercise program and better tolerability of chemotherapy was suggested in a retrospective study of 184 patients, of whom only 31 patients had lung cancer [9].

## Figures and Tables

**Table 1:** Assessments at the time of screening, at baseline, and during radiation therapy.

	Screening	Baseline	End of Week 1 of Radiation Therapy	End of Week 5 of Radiation Therapy
Demographics	X			
Medical history and medication		X	X	X
Patient and tumor characteristics		X		
Performance status		X	X	X
Assessment of distress		X		
Assessment of pain		X		
Assessment of fatigue		X		
Details of radiation therapy	X			
Radiation therapy performed as planned?			X	X
Written informed consent given	X			
Fulfillment of inclusion criteria	X			
Expected non-compliance	X			
Wear-time of the smartphone			X	X
Number of steps per day			X	X
Handling smartphones		X		
Satisfaction with the “Step Reminder” app				X
Influence of using health technology				X

## Data Availability

This article reports a trial protocol and no results. More information regarding the protocol is given at clinicaltrials.gov (NCT07627022), registered on 3 June 2026.

## Abbreviations

NSCLC	Non-small-cell lung cancer
OHG	Offene Handelsgesellschaft
SCLC	Small-cell lung cancer
